# Natural variants of human SARM1 cause both intrinsic and dominant loss-of-function influencing axon survival

**DOI:** 10.1038/s41598-022-18052-8

**Published:** 2022-08-16

**Authors:** Mirlinda Ademi, Xiuna Yang, Michael P. Coleman, Jonathan Gilley

**Affiliations:** Department of Clinical Neurosciences, John van Geest Centre for Brain Repair, University of Cambridge, Forvie Site, Robinson Way, Cambridge, CB2 0PY UK

**Keywords:** Cell death in the nervous system, Cellular neuroscience, Molecular neuroscience

## Abstract

SARM1 is a central executioner of programmed axon death, and this role requires intrinsic NAD(P)ase or related enzyme activity. A complete absence of SARM1 robustly blocks axon degeneration in mice, but even a partial depletion confers meaningful protection. Since axon loss contributes substantially to the onset and progression of multiple neurodegenerative disorders, lower inherent SARM1 activity is expected to reduce disease susceptibility in some situations. We, therefore, investigated whether there are naturally occurring *SARM1* alleles within the human population that encode SARM1 variants with loss-of-function. Out of the 18 natural SARM1 coding variants we selected as candidates, we found that 10 display loss-of-function in three complimentary assays: they fail to robustly deplete NAD in transfected HEK 293T cells; they lack constitutive and NMN-induced NADase activity; and they fail to promote axon degeneration in primary neuronal cultures. Two of these variants are also able to block axon degeneration in primary culture neurons in the presence of endogenous, wild-type SARM1, indicative of dominant loss-of-function. These results demonstrate that SARM1 loss-of-function variants occur naturally in the human population, and we propose that carriers of these alleles will have different degrees of reduced susceptibility to various neurological conditions.

## Introduction

Impairment of axons is a common pathological denominator across a wide range of neurological diseases, and maintaining axonal homeostasis is a constant challenge^1,2^. Axonal health is highly dependent on the homeostasis of nicotinamide adenine dinucleotide (NAD) and related metabolites and involves a fine interplay between pro-survival and pro-degenerative cues. In particular, the antagonistic effects of the NAD-synthesising activity of nicotinamide mononucleotide adenylyltransferase 2 (NMNAT2) and the NAD-consuming enzyme sterile alpha and TIR motif-containing protein 1 (SARM1) are critical^3–7^. Nicotinamide mononucleotide (NMN), the substrate of NMNAT activity, and NAD are respectively an activator and inhibitor of the multifunctional glycohydrolase (NAD(P)ase) activity encoded by the SARM1 TIR domain, and they regulate this activity by competing for binding to an allosteric site in the SARM1 ARM domain^8–12^. In healthy axons, NMNAT2 keeps NMN levels low, relative to NAD, to restrain SARM1 NADase activity. However, as a very labile protein, NMNAT2 is rapidly depleted in axons damaged by injury or various other stresses leading to rising NMN and declining NAD^3,13^, activation of SARM1^8,11^, and a self-reinforcing decline in NAD that represents one possible cause of degeneration. A lack or bypass of NMNAT-dependent restraint of SARM1 also appears to promote neuronal cell death in some situations^14–18^.

Animal models have implicated NMNAT-sensitive/SARM1-dependent programmed axon degeneration and/or cell death as contributing to several neurodegenerative diseases, including chemotherapy- and diabetes-induced peripheral neuropathy^19–22^, traumatic brain injury^23,24^, glaucoma^25–28^, and TDP-43 associated amyotrophic lateral sclerosis (ALS)^29^, among others^2^. Evidence for direct genetic association with human disease is more limited but is increasing. Direct causation is strongly implied for biallelic *NMNAT2* loss-of-function (LoF) mutations in three rare polyneuropathies that broadly resemble equivalent mouse models^30–34^, and for *NMNAT1* mutations that cause photoreceptor loss in Leber congenital amaurosis via a mechanism involving SARM1^16^. There is also growing evidence that SARM1 plays an important role in ALS and related motor disorders. Genome-wide association studies (GWAS) previously linked the *SARM1* chromosomal locus to sporadic ALS^35,36^ and several *SARM1* alleles encoding pro-degenerative SARM1 gain-of-function (GoF) variants with constitutively hyperactive NADase have more recently been strongly implicated as risk alleles in these diseases^17,37^.

In addition to the *NMNAT2* LoF and *SARM1* GoF alleles that have been linked to human neurodegenerative phenotypes, we predicted that other function-altering alleles for these genes would exist among the extensive genetic variation within the human population listed in online databases such as the NCBI Single Nucleotide Polymorphism Database (dbSNP) and the Genome Aggregation Database (gnomAD). We have investigated whether *SARM1* alleles encoding LoF variants, with respect to NADase activity and normal pro-degenerative function, exist in the general population. Crucially, *Sarm1* is haploinsufficient in mice, with loss of one allele resulting in a significant reduction in NADase activity and significantly delaying programmed axon degeneration triggered by injury and other stresses^38,39^. As such, it is anticipated that *SARM1* alleles encoding full LoF variants might confer meaningful axon protection even in heterozygous individuals. Furthermore, some artificial SARM1 mutants have been reported to have apparent dominant-negative effects^5,40,41^ and if natural *SARM1* alleles encoding variants with similar properties were found then heterozygous individuals could even possess a robust axon protective phenotype comparable to that associated with a complete absence of SARM1 in mice^4,5^. Here, we have identified 10 rare, naturally occurring *SARM1* alleles that show LoF in all primary assays performed, of which two were also found to have dominant LoF effects. These findings have intriguing implications in relation to individual susceptibility to human neurodegenerative axonopathies.

## Results

### Selection of naturally occurring human SARM1 variants with potential for LoF

**Figure 1:**
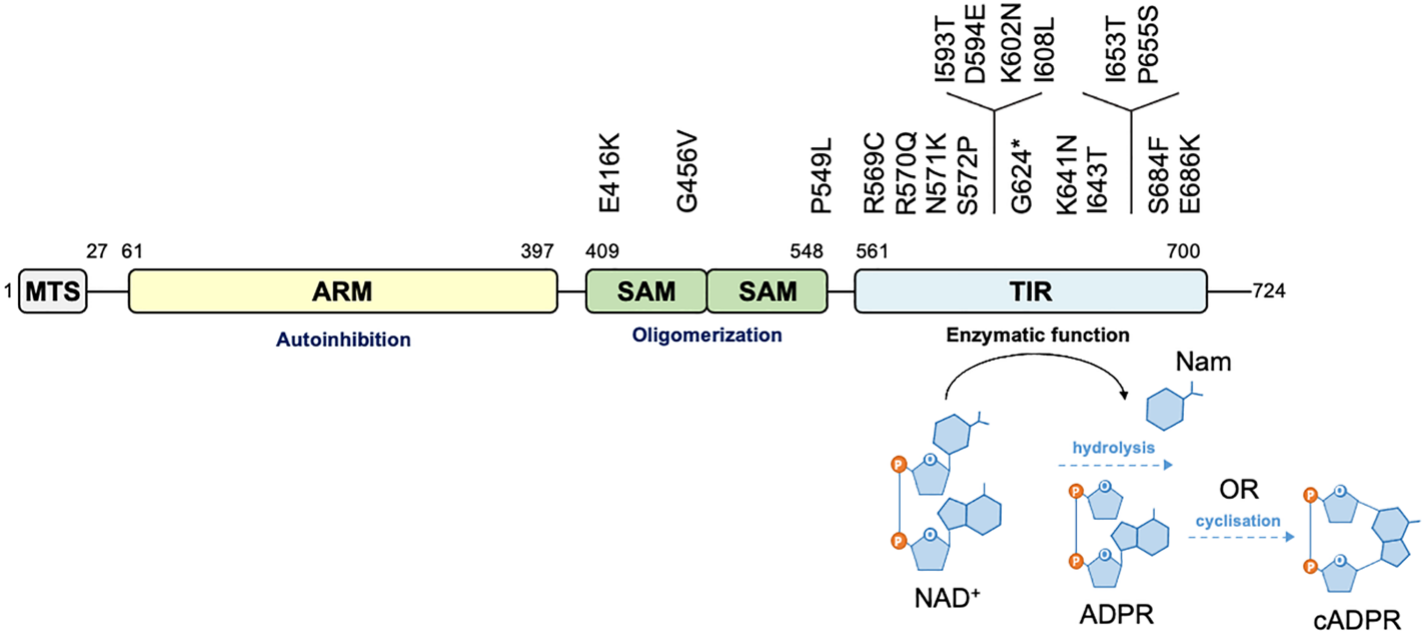
SARM1 SAM and TIR domain coding variants assessed in this study. Domain architecture highlighting important structures in the 724 amino acid human SARM1 protein showing the locations of the missense and nonsense SAM and TIR domain variation encoded by selected *SARM1* alleles. MTS, “mitochondrial” targeting sequence; ARM, HEAT/Armadillo motif domain; SAM, sterile alpha motif domains; TIR, Toll/Interleukin receptor domain.

We hypothesised that naturally occurring SARM1 coding variation that confers LoF exists in the general human population. Previous studies have shown that disruption of the SAM multimerization domains or catalytic TIR domain of SARM1 is more likely to cause protective LoF than disruption of the ARM domain, which mostly causes pro-degenerative GoF^5,7,9–11,17,37,40,42–44^. Therefore, from the more than 100 natural *SARM1* alleles listed in the dbSNP and gnomAD databases that encode SAM or TIR variants, we selected a total of 17 missense variants and one nonsense variant that we considered had good potential for LoF based on several criteria: overlap or proximity to a previously reported (artificial) inactivating mutation in the NADase catalytic pocket^7,42^, evolutionary conservation of the consensus residue, physicochemical consequences of the amino acid changes, and allele frequency (Fig. 1 and Table 1).

**Table 1:** SARM1 SAM and TIR domain coding variants in gnomAD and dbSNP.

SARM1 domain	Coding variant^a^	rsID	Gen. location and change (GRCh38)	MAF^b^
SAM	E416K	rs147156050	17:28,384,513 G > A	0.00058
	G456V	rs140340827	17:28,384,903 G > T	0.00066
	P549L	rs1241475032	17:28,388,189 C > T	0.000020
TIR	R569C	rs571724138	17:28,388,248 C > T	0.000046
	R570Q	rs539229444	17:28,388,252 G > A	0.000085
	N571K	rs150052156	17:28,388,256 C > G	0.000013
	S572P	rs781797528	17:28,388,257 T > C	0.000013
	I593T	rs782196205	17:28,388,394 T > C	0.000013
	D594E	rs782407924	17:28,388,398 T > A	–*
	K602N	rs35201495	17:28,388,422 G > T	0.000079
	I608L	rs1490414328	17:28,388,438 A > C	–**
	G624*	rs141324431	17:28,388,486 G > T	0.000033
	K641N	rs1344370780	17:28,388,539 G > T	0.000013
	I643T	rs1224037543	17:28,395,909 T > C	–**
	I653T	rs782525385	17:28,395,939 T > C	0.0000066
	P655S	rs776939252	17:28,395,944 C > T	0.000046
	S684F	rs782256561	17:28,396,162 C > T	0.000033
	E686K	rs1460394367	17:28,396,167 G > A	0.000013

*gnomAD 2.1_exome MAF of 0.000037.

**TOPMED MAF of 0.0000038.

^a^Numbering according to the canonical 724 amino acid long human SARM1.

^b^Minor allele frequency referring to the gnomAD 3.1 population except where indicated otherwise.

### Several natural SARM1 TIR domain variants do not deplete NAD in transfected HEK 293T cells

**Figure 2:**
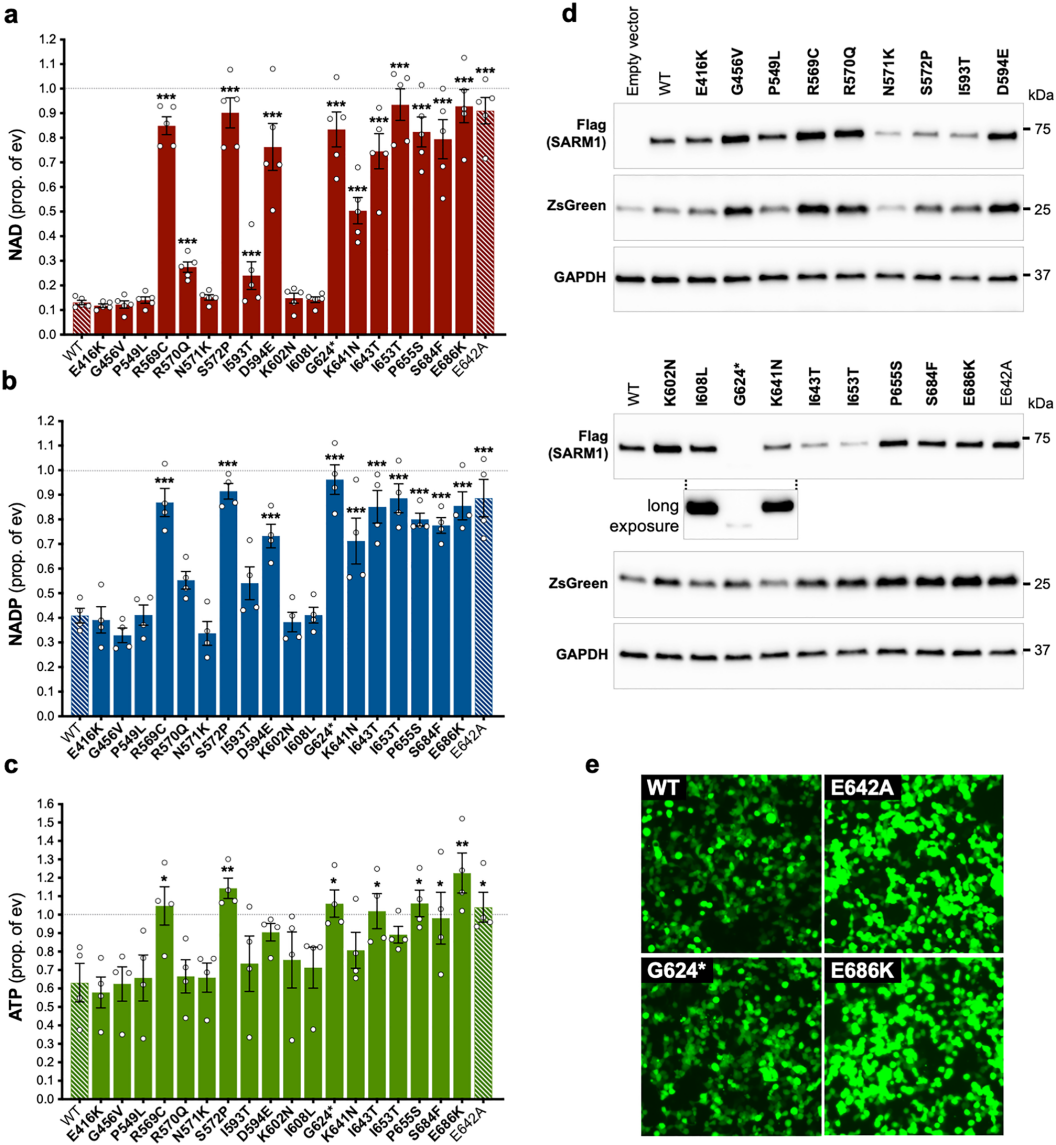
Several SARM1 TIR domain variants do not deplete NAD in transfected HEK 293T cells. (**a**,**b**,**c**) Endogenous levels of NAD (**a**), NADP (**b**), and ATP (**c**) were assessed in extracts from HEK 293T cells 24 h after transfection with 1 μg of a bicistronic construct co-expressing WT or variant SARM1 together with ZsGreen. Values are shown relative to those from cells transfected with empty vector (ev) control only (set at one, dashed black line). Means ± SEM with individual data points (from n = 4 or 5 experiments) are plotted. *p < 0.05, **p < 0.01 and ***p < 0.001, multiple pairwise comparisons to WT SARM1 with FDR correction (after log transformation of data in part **a**). Bars for the positive and negative controls (respectively WT SARM1 and the enzyme-dead E642A SARM1 artificial mutant) are hatched to differentiate them from the selected variants (bold text, filled bars). (**d**) Representative immunoblots of transfected HEK 293T cell extracts. Blots were probed with a Flag antibody to detect Flag-tagged SARM1 protein (~ 70 kDa), and antibodies against co-expressed ZsGreen (~ 26 kDa) and GAPDH (~ 36 kDa, acting as a loading control). A long exposure of the Flag antibody blot for the lower panel of variants, showing just the G624* SARM1 and flanking lanes, is included to demonstrate that G624* SARM1 is expressed but at a very low level relative to other variants. (**e**) Micrographs showing ZsGreen expression in HEK 293T cells transfected with constructs co-expressing the selected SARM1 variants as shown. While the intensity of ZsGreen fluorescence differed depending on the activity of the SARM1 variant being expressed, the majority of cells were transfected (ZsGreen positive) in each case. Differences in SARM1 (and ZsGreen) expression detected in part **d** are therefore not the result of substantial differences in transfection efficiency and was true for all variants, not just those shown.

We first tested the SARM1 SAM and TIR domain variants encoded by the selected *SARM1* alleles for potential NADase LoF by assessing their ability to deplete NAD in transfected HEK 293T cells. This established assay provides an indirect measure of the activity of exogenously-expressed Flag-tagged SARM1 variants and has the potential to detect either NADase GoF or LoF^17^. However, here we used conditions where full-length wild-type (WT) SARM1 robustly consumes NAD (due to high overexpression of a low, constitutive NADase activity) to optimise the detection of NADase LoF (Fig. 2a).

While a number of the selected variants behaved like WT SARM1 in these assays, several of the natural TIR domain variants—R569C, S572P, D594E, G624*, I643T, I653T, P655S, S684F, and E686K SARM1—replicated the effects of E642A SARM1, an artificial SARM1 mutant deficient for NADase activity^7^, by failing to deplete NAD significantly (Fig. 2a). These were thus predicted to be variants with substantial NADase LoF. K641N SARM1 had an intermediate effect on metabolite levels indicative of moderate LoF, while a slight reduction in NAD consumption by R570Q and I593T SARM1 could be suggestive of very modest LoF. Consistent with our previous findings^17^, the variants with the strongest putative NADase LoF also failed to deplete NADP, an NAD analogue and alternative substrate of SARM1 glycohydrolase activity^12,42,45,46^, and ATP, whose depletion is a likely lagging consequence of the NAD loss (Fig. 2b,c).

Importantly, immunoblotting indicated that reduced NAD depletion in the transfected HEK 293T cells was, in most cases, not simply a result of lower SARM1 expression (Fig. 2d). In fact, the majority of the putative LoF variants were found to be expressed at levels similar to, or higher than WT SARM1. Furthermore, even in those cases where expression was found to be noticeably lower, the degree to which expression was reduced would, with the exception of I593T and G624* SARM1, probably not be sufficient by itself to account for the observed lack of NAD depletion. In the case of G624* SARM1, its expression is barely detectable in these assays (Fig. 2d), and this likely reflects reduced stability of the truncated protein rather than less efficient transfection (Fig. 2e). This very low expression would clearly compromise its ability to deplete cellular NAD if it possessed significant NADase activity but, crucially, G624* SARM1 lacks key catalytic residues due to C-terminal truncation of the TIR domain, including the essential E642 residue, and is thus anyhow predicted to be full NADase LoF.

Interestingly, levels of co-expressed ZsGreen in the transfected HEK 293T cells were found to vary and this was largely independent of transfection efficiency (Fig. 2d,e). This could, in part, be explained by a model in which NAD depletion caused by overexpression of NADase-competent SARM1 variants suppresses de novo protein synthesis^17,47^. In this model, cells with substantial NAD depletion express lower levels of ZsGreen than cells where NAD levels remain high. However, there are some exceptions. In particular, ZsGreen levels are higher than expected in cells expressing G456V or K602N SARM1 in light of the substantial NAD depletion seen. This could be the result of a subtle LoF resulting in slower NAD depletion compared to WT SARM1 (but still fast enough to cause maximal depletion by the assay endpoint) allowing for a longer period of ZsGreen expression (and SARM1 expression as well). In addition, the consistently low ZsGreen expression from empty vector is likely a transcriptional or translational consequence of an empty first cistron in the bicistronic expression cassette and thus completely independent of NAD levels.

### Several natural human SARM1 TIR domain variants lack constitutive and NMN-induced NADase activity

As measurements of NAD levels in transfected HEK 293T cells only provide an indirect measure of NADase activity of the exogenous SARM1 variants, we next directly tested the activity of recombinant SARM1 variant proteins using an established method where Flag-tagged SARM1 is purified from HEK 293T cell lysates by immunoprecipitation^17^.

**Figure 3:**
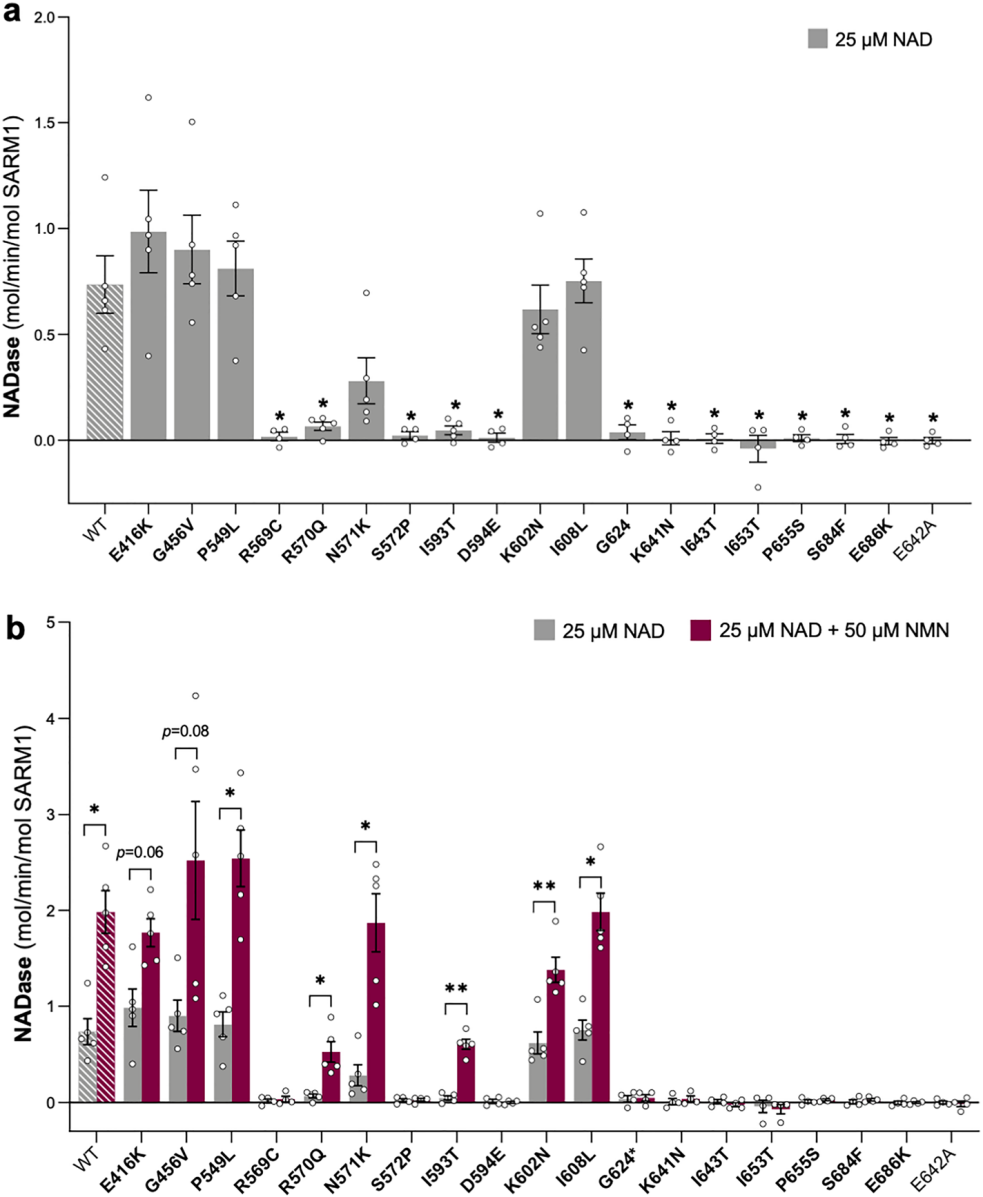
Several SARM1 TIR domain variants are LoF for basal and NMN-induced NADase activity. (**a**) Basal NADase activity of recombinant WT SARM1, enzyme-dead E642A (representing a full LoF a control), and naturally occurring variant SARM1 in the presence of 25 μM NAD alone. Values are depicted as means ± SEM with individual data points (n = 4 or 5) plotted. *p < 0.05, multiple pairwise comparisons to WT SARM1 with FDR correction. (**b**) Comparison of basal and induced NADase activities of SARM1 variants in the presence of 25 μM NAD with or without the addition of 50 μM NMN. Constitutive activities (no NMN) are as in part **a** with rates + NMN calculated from assays performed in parallel. Data are presented as means ± SEM with individual data points (n = 4 or 5) plotted. *p < 0.05 and **p < 0.01, multiple paired t-tests with FDR correction of basal (NAD alone) versus induced (NAD + NMN) for each variant. Rate calculations are described in detail in the Materials and Methods. Rate measurements are determined as the difference between NAD levels in the original reaction and at the reaction endpoint. Endpoint NAD levels for inactive variants should, in theory, be identical to the starting level, but fluctuations on either side of the starting value due to pipetting variation mean that small negative rates can be obtained using this method. As in Fig. 2, bars for controls in parts a and b are hatched to differentiate them from variants (bold text, filled bars).

We first measured the constitutive activity of equal amounts of each purified protein in the presence of 25 μM NAD alone (Fig. 3a). We obtained an NAD consumption rate for WT SARM1 that was comparable to that reported previously using the same assay^17^ and found that all SARM1 variants that were strong or moderate LoF for depletion of NAD in transfected HEK 293T cells—R569C, S572P, D594E, G624*, K641N, I643T, I653T, P655S, S684F, and E686K SARM1—also had little or no activity in these recombinant protein assays. The lack of activity of these variants matched that of NADase-dead E642A SARM1 suggesting complete LoF for constitutive NADase. Unexpectedly, R570Q and I593T SARM1, both of which only showed a very modest LoF effect in transfected HEK 293T cells, appeared to be full LoF for basal NADase activity in these assays. All other variants had activities similar to WT SARM1, consistent with their ability to deplete NAD in transfected HEK 293T cells, apart from N571K SARM1 which was found to have a moderately reduced NAD consumption rate.

We next tested the NADase activity of each SARM1 variant in the presence of NMN, a physiological activator of SARM1 NADase during axon degeneration^8,11,13,18,48^. Reactions contained 25 μM NAD together with 50 μM NMN, a concentration of NMN that has previously been shown to reliably induce the NAD consumption rate of recombinant WT SARM1 to around three times that of its basal activity in these assays^17^. Notably, all the SARM1 variants that were found to be both moderate or strong LoF for NAD depletion in transfected HEK 293T cells and full LoF for constitutive NADase activity—R569C, S572P, D594E, G624*, K641N, I643T, I653T, P655S, S684F, and E686K SARM1—also lacked NADase activity in the presence of NMN (Fig. 3b). This was comparable to enzyme-dead control E642A SARM1 and is entirely consistent with complete loss of TIR domain catalytic activity in these variants. Intriguingly, despite having no constitutive NADase activity, R570Q and I593T SARM were found to have some activity in the presence of NMN indicating that they are not enzymatically dead. In contrast, all of the variants with a WT-like constitutive NADase activity, as well as N571K SARM1, showed increased levels of activity in the presence of NMN, indicating that none are LoF for NMN-inducibility.

Notably, the complete lack of constitutive NADase activity for R570Q and I593T SARM1, and to a lesser extent the relatively low constitutive activity of N571K SARM1, is difficult to reconcile with the extent to which they can deplete NAD in transfected HEK 293T cells (compare Figs. 2a and 3a). While recombinant R570Q and I593T SARM1 have detectable NADase activity in the presence of NMN, there is as yet no evidence that NMN-induced activity contributes significantly to cellular NAD depletion in transfected HEK 293T cells. Besides, the NMN-induced activities of recombinant R570Q and I593T SARM1 are anyhow much lower than that of WT SARM1. Instead, it is possible that our recombinant protein assays do not reflect the true NADase activities of these particular variants. While this could equally apply to any variant, it is notable that, unlike R570Q and I593T SARM1, the others lacking constitutive NADase activity in the assay (including neighbouring variants R569C, S572P, and D594E SARM1) also lack NMN-induced activity and do not substantially deplete NAD in transfected HEK 293T cells, consistent with *bona fide* LoF.

### Natural SARM1 TIR domain variants with confirmed full NADase LoF are unable to promote injury-induced neurite degeneration

Axons of neurons lacking SARM1 are strongly resistant to degeneration for several days after injury and other stresses, but re-expression of functional SARM1 restores the degenerative phenotype^4,5^. We, therefore, compared the extent to which exogenous expression of WT SARM1 or our selected SAM and TIR domain SARM1 variants could restore normal injury-induced neurite degeneration in primary neuronal cultures of *Sarm1*^*-/-*^ neurons. We used an established technique where mouse superior cervical ganglion (SCG) neurons in primary cultures are microinjected with controlled amounts of expression constructs, including an expression construct for fluorescent DsRed protein for visualisation of the injected neurons and their neurites^49^.

**Figure 4:**
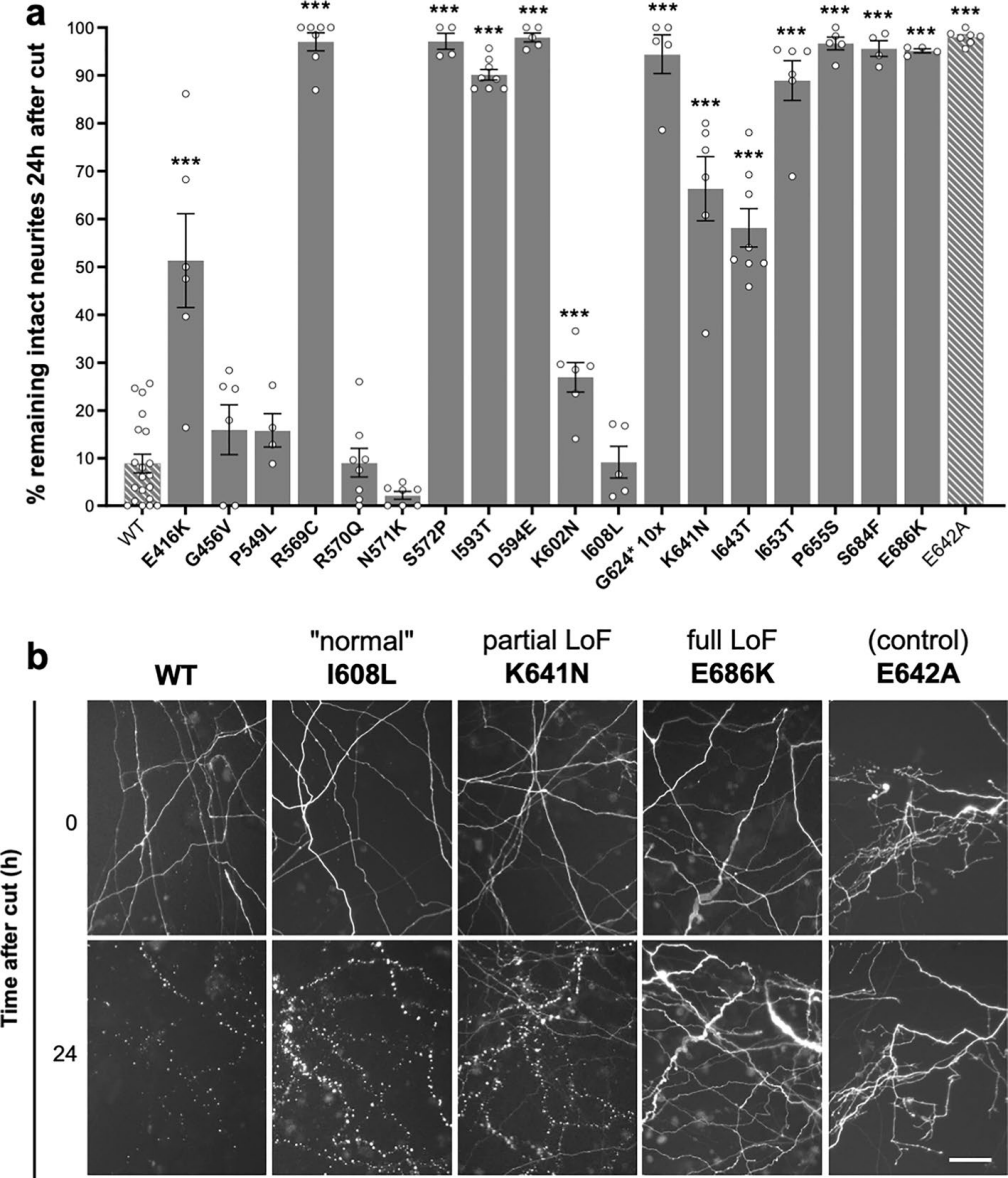
NADase LoF SARM1 TIR domain variants are unable to promote injury-induced neurite degeneration. (**a**) *Sarm1*^-/-^ SCG neurons were microinjected with 2.5–5 ng/μl of SARM1 expression constructs (pCMV-Tag4 vector backbone) encoding untagged WT or variant/mutant human SARM1, together with 40 ng/μl pDsRed2 encoding DsRed to allow for visualisation of injected neurites. DsRed-labelled neurites were transected 48 h after injection. Degeneration was quantified by determining the percentage of intact DsRed-labelled neurites remaining 24 h after transection compared to at the time of cut (0 h). Data are presented as means ± SEM with individual data points plotted (n = 4–20), each representing average neurite survival from independent assays of multiple injected neurons. ***p < 0.001, multiple pairwise comparisons to WT SARM1 with FDR correction. Cut neurites in pDsRed2 only control injections did not undergo degeneration (~ 94.0 ± 1.2% neurite survival at 24 h). (**b**) Representative fluorescence images, as used for the quantification shown in part **a**, of transected, DsRed-labelled neurites of SARM1-deficient neurons injected with constructs expressing WT or variant/mutant SARM1 (as indicated) at 0 and 24 h after cut used for the quantification shown in part **a**. Controls (wild-type and enzyme-dead E642A SARM1) and three exemplary phenotypes are shown: I608L SARM1 resembling wild-type SARM1 ("normal"), K641N SARM1 displaying a partial LoF phenotype, and E686K SARM1 displaying full LoF. Scale bar, 50 μm. Separate immunostaining assays revealed that each variant, except for G624*, is expressed at a broadly similar level to WT SARM1 in the cell bodies of injected SCG neurons (Supplementary Fig. 1a) and immunoblotting of extracts of transfected HEK 293T cells confirmed that SARM1 variants of the expected size are expressed from the specific expression constructs used in the injection experiments (Supplementary Fig. 1b).

Induction of SARM1 enzyme activity by NMN is required to trigger axon degeneration^8,11,18^. Consistent with this, we found that most of the natural SARM1 variants that responded to NMN like WT SARM1 in our recombinant protein activity assays robustly restored degeneration of transected *Sarm1*^*-/-*^ SCG neurites, whereas enzyme-dead E642A SARM1 and the natural SARM1 variants with full NADase LoF did not (Fig. 4a,b).

Intriguingly, two variants with apparent WT-like NADase activity in the recombinant protein assays, E416K and K602N SARM1, showed partial LoF in the neurite degeneration assays, while two variants with presumed complete NADase LoF, K641N and I643T SARM1, appeared to be partially functional. Furthermore, of the variants that robustly depleted NAD in transfected HEK 293T cells but were not fully functional for NADase activity in our recombinant protein assays, R570Q and N571K SARM1 fully restored neurite degeneration, consistent with normal pro-degenerative function, whereas I593T SARM1 behaved like a full LoF. While unrepresentative NADase activities obtained in our recombinant proteins assays for a few of these variants (see above) could explain some of the discrepancies, differences in expression could also play a part. For example, low expression of an NADase-competent SARM1 variant might limit its ability to restore neurite degeneration, whereas high expression of a variant with partial NADase LoF could be sufficient to restore some degeneration. Although non-quantitative immunostaining revealed that levels of these nonconforming SARM1 variants appeared broadly similar to WT SARM1 in the cell bodies of the injected neurons (Supplementary Fig. 1a), this method is not sensitive enough to rule out a functionally relevant deficiency or excess specifically within the neurites themselves.

Despite using 10 times the concentration of the G624* SARM1 expression construct in anticipation of poor expression of this truncated variant (see above), G624* SARM1 levels still appeared substantially lower than WT SARM1 in the injected neurons (Supplementary Fig. 1a). While this complicates the interpretation of its effects in this assay, we nevertheless predict that, even if expressed at WT levels, G624* SARM1 would be incapable of restoring degeneration of cut *Sarm1*^*-/-*^ neurites due to its inherent lack of NADase activity in our assays of recombinant protein.

Interestingly, we observed that exogenous expression of SARM1 was also moderately cytotoxic in these assays, again in a broadly NADase activity-dependent manner (Supplementary Fig. 2). While WT SARM1 and other NADase-competent variants caused some loss of neurons between 24 and 48 h after injection (prior to performing neurite transections), negligible cytotoxicity was seen for the NADase LoF variants. The cytotoxic effect largely mirrored variant functionality in the neurite degeneration assays, albeit the effect was weaker and more variable. Notably, we purposefully injected small amounts of SARM1 expression constructs in these assays to avoid excessive overexpression and we propose that this cytotoxicity might reflect an interaction between a moderate, acute increase in NADase activity in neurons previously lacking SARM1 and the stresses associated with injection.

### D594E and E686K SARM1 have a dominant LoF effect on neurite degeneration

SARM1 multimerises to form an octameric ring structure in solution^9,42,43,50^ and this multimerization is necessary for its pro-degenerative function^5,42^. A small number of artificial SARM1 mutants have previously been shown to exert an apparent dominant-negative effect when expressed in wild-type cultures, with the mutants presumably disrupting TIR domain interactions and NADase activity of endogenous (WT) SARM1 within hetero-oligomers to block its pro-degenerative activity and prevent axon degeneration^5,40,41^. We, therefore, investigated whether any of our naturally occurring SARM1 TIR domain variants with confirmed LoF for NADase activity and pro-degenerative function might also have dominant LoF properties.

To test this, we assessed the effects of exogenously expressed SARM1 LoF variants on degeneration in microinjected wild-type SCG primary culture neurons. We waited seven days after injection before transecting neurites to test for dominant LoF properties to allow sufficient time for the formation of heterooligomers between the exogenously expressed SARM1 and endogenous (WT) SARM1. Given that prolonged and robust expression of fluorescent proteins can be cytotoxic^3^, we injected a low concentration of a single bicistronic expression construct encoding SARM1 and ZsGreen in these experiments, rather than separate SARM1 and DsRed constructs, with the resulting relatively lower expression of ZsGreen being sufficient for visualisation over this extended timeframe without causing significant cytotoxicity.

**Figure 5:**
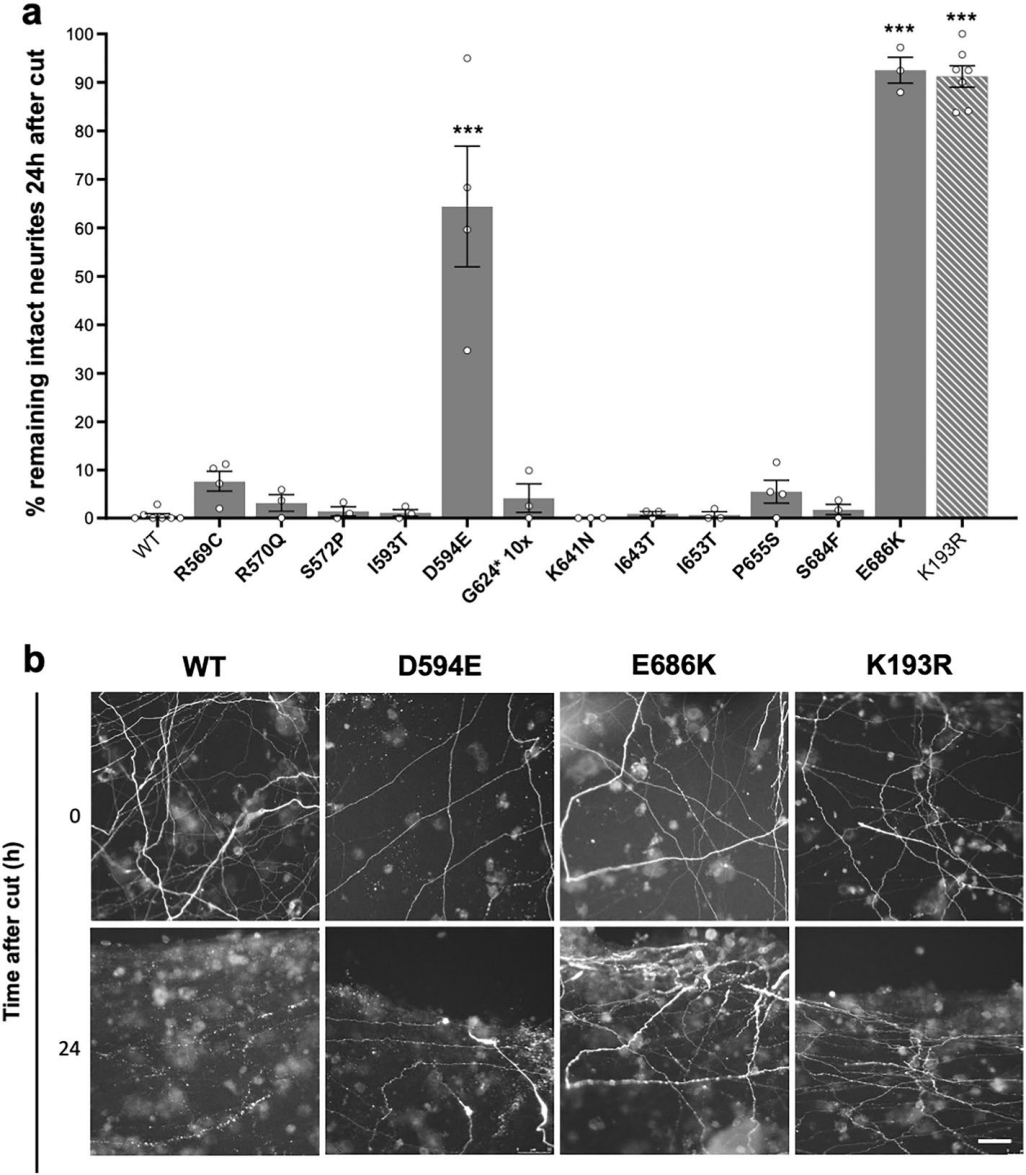
D594E and E686K SARM1 have dominant LoF properties. (**a**) Wild-type SCG neurons were injected with 5 ng/μl bicistronic expression constructs (pLVX-IRES-ZsGreen1 vector backbone) encoding WT or variant/mutant human SARM1 with a C-terminal Flag tag and ZsGreen. Co-expressed ZsGreen allowed visualisation of the neurites of the injected neurons, and these were transected 7 days after injection. Degeneration was quantified by determining the percentage of intact ZsGreen-labelled neurites remaining 24 h after transection compared to at the time of cut (0 h). Data are presented as means ± SEM with individual data points (n = 3–6). ***p < 0.001, multiple pairwise comparisons to WT SARM1 with FDR correction. (**b**) Representative fluorescence images, as used for the quantification shown in part **a**, of transected ZsGreen-labelled neurites of neurons injected with constructs expressing the indicated SARM1 variants at 0 and 24 h after the cut. Scale bar, 50 μm.

K193R SARM1, a confirmed dominant-negative mutant^41^, showed a strong phenotype under these conditions, protecting all neurites for up to 24 h after injury, thereby establishing it as a valid method for screening (Fig. 5). Most of our natural variants did not block the normal, rapid degeneration of injured, wild-type neurites, but two, D594E and E686K SARM1, were found to have clear dominant LoF properties, with E686K protecting injured neurites as robustly as K193R SARM1, and D594E having only a slightly weaker effect (Fig. 5). Individuals that are heterozygous for the alleles encoding D594E or E686K SARM1 are thus predicted to show stronger protection against programmed axon degeneration than those with intrinsic (non-dominant) LoF.

## Discussion

Here, we have identified 10 naturally occurring human *SARM1* alleles encoding SARM1 TIR domain missense or nonsense variants that are clear LoF in all the tests employed in this study. These 10 variants—R569C, S572P, D594E, G624*, K641N, I643T, I653T, P655S, S684F, and E686K SARM1—fail to deplete NAD in transfected HEK 293T cells, they lack NADase activity even in the presence of the physiological activator NMN, and they fail to promote normal degeneration of neurites in primary SCG neuron cultures. Of these, D594E and E686K SARM1 have dominant LoF properties as they can block neurite degeneration even in the presence of endogenous (WT) SARM1. We predict that individuals heterozygous for either of the two dominant LoF variants will possess axons that could approach the strong resistance to degeneration similar to that seen in mice completely lacking SARM1^4,5^, provided that physiological levels of expression confer as robust a dominant negative effect. Individuals that are heterozygous for intrinsic (non-dominant) LoF will likely have a more modest, but still meaningful axon protection phenotype given that *Sarm1* haploinsufficiency provides some degree of protection against programmed axon degeneration triggered by injury and other stresses in mice and primary neurons^39^. As such, carriers of these alleles may have varying degrees of reduced susceptibility to a variety of neurological conditions.

There is already evidence that *SARM1* alleles encoding variants with altered function are potential modifiers of disease risk, likely altering penetrance or expressivity. Specifically, we and others have previously reported that *SARM1* alleles encoding ARM domain variants with constitutively hyperactive NADase and enhanced pro-degenerative capacity are enriched in individuals with ALS and related motor nerve disorders^17,37^. A relative paucity of carriers of *SARM1* alleles encoding LoF variants among individuals with these disorders compared to the general population might therefore also be predicted, although confirmation of this has not yet been possible due to a combination of the low frequency of our confirmed LoF alleles and the relatively restricted size of currently available patient cohorts. It is also likely that any protective effect would only be detectable in a population where disease is expected, for example by modifying age-of-onset in carriers of fully penetrant pathogenic mutations. Nevertheless, we expect that this will become possible as more *SARM1* LoF alleles are identified, and as disease cohorts expand. This should also facilitate identifying other potential links between *SARM1* variant alleles and other neurological disorders where SARM1 is predicted to play an important role. Importantly, any association of natural *SARM1* LoF alleles with the absence or delayed onset of disease would support the notion that drugs blocking programmed axon death could be effective in that disorder.

The confirmed LoF variants reported in this study are all rare, with ~ 0.04% of the general population being a carrier of one of these alleles based on the sum of their individual MAFs (Table 1). However, we have restricted our classification of confirmed LoF variants to just those that show clear LoF in all tests employed here. Interestingly, other variants among our original group of 18 also showed some degree of LoF in just one or a subset of the assays. While the varying degrees of NADase LoF determined for recombinant R570Q, N571K and I593T SARM1 might not be representative of their real activities (as discussed above), the failure of E416K, I593T and K602N SARM1 to fully restore degeneration of SCG neurites could reflect real LoF phenotypes with functional relevance in carrier individuals. Our strict classification might thus underestimate the number of LoF variants among our original group. In fact, ~ 0.17% of the general population are carriers of an allele encoding a SARM1 variant that shows any degree of LoF in our neurite degeneration assay. Furthermore, we have functionally tested just a small proportion of all the existing natural missense or nonsense variation within the SARM1 SAM and TIR domains, with a high probability that other LoF alleles exist among the remainder. In addition, there are naturally occurring splice and frameshift variants in gnomAD encoding SARM1 variants lacking the catalytic TIR domain that are also predicted to be full LoF alleles. As such, the real number of carriers of *SARM1* LoF alleles is likely to be at least ~ 0.2–0.3%, and possibly substantially higher, which would be enough to have a meaningful impact on axon health at the population level.

Given that mice lacking SARM1 are overtly normal, even into old age^51,52^, the predicted relatively low frequency of *SARM1* LoF alleles among the general population is perhaps somewhat surprising. However, the extent to which the *SARM1* gene has been evolutionarily conserved argues that there must be significant selective pressure to retain SARM1 functionality. This could either reflect that the alternative function of SARM1 in innate immunity is essential^53^, something that would not be challenged in mice housed in a pathogen-free environment, or that axon protection resulting from SARM1-deficiency confers an as yet unidentified subclinical phenotype that only impacts survival in the wild.

**Figure 6:**
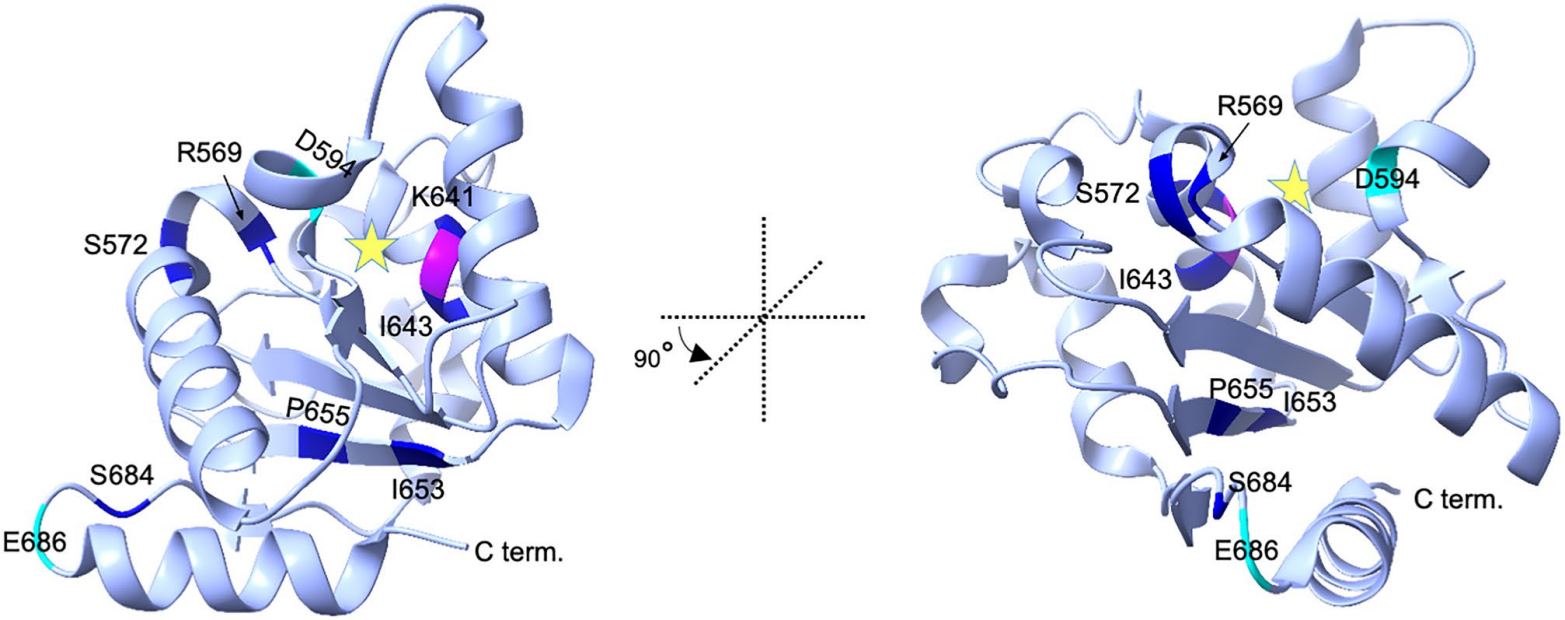
Locations of missense variation that confer NADase LoF in the SARM1 TIR domain crystal structure. Ribbon diagram of the reported crystal structure of the human SARM1 TIR domain showing the locations of residues that are altered by the natural missense coding variants identified in this study to cause LoF. The structure presented is based on PDB ID:6O0R (bound to ribose) with the position of the predicted NAD substrate-binding pocket indicated by a star. Two views are shown differing by a rotation of 90° anti-clockwise along the vertical axis. The locations of residues that are altered to confer basic LoF are shown in dark blue and those that are altered to confer dominant LoF properties are shown in cyan. The catalytic E642 residue is labelled magenta. ChimeraX (http://www.rbvi.ucsf.edu/chimerax/) was used to generate the structures shown.

All the full LoF variants identified here encode changes in the SARM1 catalytic TIR domain, but they are widely distributed within the domain (Fig. 6). Several overlap with, or are in proximity to residues that are already known or predicted to be involved in catalysis and/or substrate binding, such as F565-R570, D594, and E642. Others are close to other functionally-important residues, including H685, that may modify TIR domain conformation to alter activity^42,46^. Interestingly, E686K variant SARM1 changes a residue that has recently been shown to be important for an autoinhibitory intra-protomer interaction between the ARM and TIR domains within the SARM1 octameric ring structure that normally restricts TIR-TIR interactions to restrain catalytic activity^9,43,44^. If the E686K change stabilises the ARM-TIR interaction then it could represent an alternative LoF mechanism. However, an artificial E686A mutation actually increases NADase activity^44^, and switching a negative charge to a positive charge at this residue would arguably cause greater disruption of the ARM-TIR interaction and greater GoF. Instead, we postulate that E686K causes a change within the TIR domain itself resulting in an inherently catalytically inactive SARM1 variant that would negate any potential GoF effect relating to the ARM-TIR interface.

While many SARM1 variants/mutants with loss of enzyme activity have been identified, very few also have dominant LoF effects. Furthermore, loss of enzyme activity itself is not an absolute requirement for dominant LoF effects as dominant LoF mutant K193R SARM1 retains basal enzyme activity. Instead, dominant LoF variants/mutants may share an ability to prevent the NMN binding-dependent conformational changes that would otherwise result in the pro-degenerative activation of enzyme activity of any WT SARM1 protomers within the hetero-oligomers. This would presumably occur via a combination of intra- and inter-protomer interactions within the octameric structure and, in this respect, the involvement of the E686 residue in one of the ARM-TIR interactions could be important in relation to the dominant LoF effects of E686K SARM1. The amino acid changes in the strong dominant LoF E686K SARM1 and weaker D594E SARM1 identified here are close to the previously reported strong and weaker dominant-negative changes, H685Y and K597E^40,41^, further highlighting these regions as being mechanistically interesting. However, there seems to be no obvious relationship between these two regions, or the location of K193, within the SARM1 structure. The way in which the dominant LoF variants/mutants in each separate region confer their effects may thus be different.

In summary, we show that multiple coding variants encoded by natural human *SARM1* alleles impair the ability of SARM1 to drive axon degeneration. Together with previous data showing that *Sarm1* haploinsufficiency confers partial protection in a range of disease models^39^, this suggests that carriers of these alleles could be at reduced risk of a number of neurological disorders. The combined frequency of LoF alleles is likely to be high enough to have a meaningful impact on population health, particularly when taken together with predicted LoF based on other nonsense, frameshift and splicing alleles, and association of *SARM1* LoF alleles with low risk or delayed onset of any disorder would support the use of SARM1-blocking drugs in those cases.

## Methods

All methods were carried out in accordance with relevant guidelines and regulations.

### Generation of constructs

Human SARM1 coding variants were selected from the publicly available dbSNP and gnomAD (v3.1) databases at https://www.ncbi.nlm.nih.gov/snp/ and gnomAD (v3.1) and https://gnomad.broadinstitute.org/, respectively. Sequence alignment of SARM1 orthologues to assess evolutionary conservation of residues was performed using Clustal Omega. SARM1 variant expression constructs were generated by introducing the desired point mutations into the complete open reading frame (ORF) of the canonical 724 amino acid long human SARM1 (NCBI Protein Reference Sequence: NP_055892.2) using an adapted QuikChange® II site-directed mutagenesis protocol (Stratagene), as previously described^17^. Successful mutagenesis and the absence of PCR errors was confirmed by DNA sequencing (Department of Biochemistry, University of Cambridge). C-terminal Flag-tagged SARM1 variants were expressed from a pLVX-IRES-ZsGreen1 vector backbone (Takara Bio) (alongside ZsGreen expressed from its bicistronic cassette) in all experiments except those described in Fig. 4 where untagged SARM1 variants were expressed from a pCMV-Tag4 vector backbone (Agilent Technologies). pEGFP-C1 vector (Clontech) expressing enhanced green fluorescent protein (eGFP) was used as a transfection control for the SARM1 pCMV-Tag4-based expression constructs. pDsRed2-N1 vector (Clontech) expressing Discosoma red fluorescent protein (DsRed2) was used to label microinjected primary neurons and their neurites.

### HEK 293T cell culture and transfection

Human embryonic kidney (HEK) 293T cells (clone 17, [HEK 293T/17]) were obtained from the American Type Culture Collection (ATCC). Cells had been authenticated by STR profiling and mycoplasma contamination was not detected. Cells were grown in DMEM with 4500 mg/L glucose and 110 mg/L sodium pyruvate (PAA), supplemented with 2 mM glutamine, 1% penicillin/streptomycin (both Invitrogen), and 10% fetal bovine serum (PAA). Transfections were performed at 70–80% confluency using Lipofectamine 2000 (Invitrogen) and Opti-MEM™ (Thermo Fisher Scientific) following the manufacturer’s protocol. The amount of DNA transfected in each experiment is indicated in the relevant figure legend or sections below.

### In-cell metabolite quantification in HEK 293T cells

24-well plates containing transfected HEK 293T cells were placed on ice and cells in individual wells collected and washed once in ice-cold PBS. Whole-cell lysates were prepared by extensively vortexing cell pellets over a 10 min period in ice-cold KHM buffer, consisting of 110 mM potassium acetate, 20 mM Hepes (pH 7.4), 2 mM MgCl_2_ 0.1 mM digitonin (all Sigma) and supplemented with cOmplete™, Mini, EDTA-free protease inhibitor cocktail (Roche). Cell lysates were cleared of insoluble material by gentle centrifugation (5 min, 3000 rpm at 4 °C). Protein concentrations in the cleared lysates were then determined using the Pierce™ BCA Protein Assay Kit (Thermo Fisher Scientific) and diluted with ice-cold KHM buffer to 0.5 μg/μl. The NAD/NADH-Glo™ and NADP/NADPH-Glo™ Assay (both Promega Biosciences) were used to measure NAD and NADP levels. 25 μl of lysate was mixed with 12.5 μl 0.4 M HCl and heated to 60 °C for 15 min before being allowed to cool to room temperature (RT) for 10 min followed by neutralisation by adding 12.5 μl 0.5 M Tris base. 10 μl of each neutralised reaction was then mixed with 10 μl of the relevant detection reagent on ice in wells of a 384-well white polystyrene microplate (Corning). Plates were incubated for 45 min at 25 °C before obtaining luminescence values using a GloMax® Explorer microplate reader (Promega Biosciences). ATP levels in lysates were measured using the CellTiter-Glo Assay (Promega Biosciences). Here, lysates were directly mixed 1:1 with the detection reagent on ice in wells of a 384-well white polystyrene microplate (Corning). Luminescence values were read in the GloMax® Explorer microplate reader after an incubation period of 15 min. All values were calculated in relation to standard curves generated from a serial dilution of the respective nucleotides. Data are expressed relative to values obtained from cells transfected with the empty vector control.

### Immunoblotting

Lysates from transfected HEK 293T cells (0.5 μg protein/μl, see above) were diluted 1:1 in 2 × SDS-PAGE loading buffer. Samples were incubated at 100 °C for 3 min and 10 μl of sample per lane resolved on 4–20% Mini-PROTEAN gradient gels (Bio-Rad) before being transferred to Immobilon-P membrane (Millipore). Blots were then blocked in 5% milk in TBS (50 mM Trizma base and 150 mM NaCl, PH 8.3, both Sigma), followed by incubation with primary antibodies diluted in TBS with 0.05% Tween-20 (Sigma) (TBS-T) and 5% milk at 4 °C overnight. Blots were then washed three times in TBS-T (5–10 min each) and incubated with the appropriate HRP-conjugated secondary antibody (1:3000, 1 h at RT, all Bio-Rad) in TBS-T with 5% milk. Blots were again washed three times in TBS-T (5–10 min each) and once in TBS before being incubated with Pierce™ ECL Western Blotting Substrate (Thermo Fisher Scientific) and imaged with an Alliance chemiluminescence imaging system (UVITEC Cambridge). Primary antibodies used were as follows: mouse ANTI-FLAG® M2 monoclonal antibody (2 μg/ml, Sigma F3165), mouse monoclonal anti-ZsGreen (1:1000, OriGene Technologies TA180002), mouse monoclonal anti-GAPDH (1:2000, Abcam, ab8245), mouse monoclonal anti-SARM1 antibody (1:2000)^54^, and mouse monoclonal anti-GFP (1:2000, Sigma 11,814,460,001). Blots (either whole or cut according to protein size) were probed either sequentially or simultaneously with different antibodies, as indicated in the uncropped immunoblot image figures included in the Supplementary information.

### SARM1 variant immunopurifications

Recombinant, C-terminal Flag-tagged SARM1 (WT or variants) was purified from transfected HEK 293T cells. Wells of a 6 well-plate, or 10 cm dishes (for SARM1 variants expressed at low levels, including S572P, G624*, I643T and I653T) were transfected with 4 μg or 24 μg of expression construct, respectively. Prior to transfection, HEK 293T cells were pre-treated with 2 mM Nicotinamide Riboside (NR), an NAD biosynthetic precursor, to boost cellular NAD levels and improve expression and yield of NADase-competent SARM1 variants.

Cells were lysed in ice-cold KHM buffer 24 h after transfection as described above but with lysates being diluted to a final concentration of 1 μg/μl. Lysates were then incubated with 50 μl/ml of washed Pierce™ Protein A/G Magnetic Beads (Thermo Fisher Scientific) together with 20 μg/ml anti-Flag M2 antibody (Merck F3165) on a rotator overnight at 4 °C. Subsequently, beads were washed three times in KHM buffer and then once in PBS (with protease inhibitors) before being resuspended in PBS containing 1 μg/μl BSA to reduce loss of SARM1 enzymatic activity when frozen for storage. Immunoblotting was used to determine the concentration of purified bead-bound SARM1 protein as described previously^17^. Of particular note, G456V SARM1 was not efficiently recognized by the SAM domain antibody used for the initial quantification (kindly provided by AstraZeneca), presumably due to disruption of the epitope. Instead, G456V SARM1 was quantified separately based on Flag immunoblotting relative to WT SARM1.

### NADase activity assays

NADase reactions were optimized to determine the ideal amount of immunoprecipitated SARM1 protein and time points to use^17^. NADase reactions were performed with the recombinant SARM1 attached to beads in 25 μl reactions (in PBS) containing 1–5 ng/μl (0.0124–0.0618 pmol/μl) purified SARM1 protein together with 25 μM NAD only, or 25 μM NAD with 50 μM NMN. Reactions were kept on ice at all points during the setup to minimize NAD consumption before starting the NADase assay at 25 °C. Reactions with bead-bound SARM1 were thoroughly mixed prior to the incubation period, as well as once or twice during the incubation. NAD consumption was measured as the difference between starting levels (0 h) and levels remaining at an endpoint that was optimised for each variant to obtain rates as close as possible to the most accurate, maximal rate under these conditions (either 1 h or 2 h for active variants, or 24 h for inactive variants).

The NAD/NADH-Glo™ assay was used to measure NAD levels in 5 μl aliquots of each reaction mix immediately after being set up (whilst still on ice to obtain precise starting levels), and at specific endpoints. Aliquots were mixed with 2.5 μl of 0.4 M HCl, to stop the reaction, followed by neutralisation with 2.5 μl 0.5 M Tris base. Samples were then diluted 1:50 in buffer containing 50% PBS, 25% 0.4 M HCl and 25% 0.5 M Tris base to bring NAD concentrations down to the linear range of the NAD/NADH-Glo™ Assay. 10 μl of the diluted samples were then mixed with 10 μl of NAD/NADH-Glo™ detection reagent on ice in wells of a 384-well white polystyrene microplate (Corning). After an incubation period of 40 min at 25 °C luminescence values were read on a GloMax® Explorer microplate reader. NAD concentrations were determined relative to an NAD standard curve.

Control immunoprecipitations using extracts from HEK 293T cells transfected with empty vector revealed that a low level of non-specific NAD-consuming activity was co-purified with the bead/antibody complexes. Whilst this non-specific activity was small, it did contribute significantly to activity rates calculated for low yield inactive variants (particularly G624* SARM1) that necessitated the use of very high bead concentrations in the reactions. As such, we calculated the non-specific NAD-consuming activity per starting volume of beads (average of n = 4 control immunoprecipitations) and subtracted this, based on the amount of beads present in each reaction, to obtain a corrected rate for each variant. NAD consumption rates were normalised to SARM1 protein concentration and are presented as mol NAD consumed per min per mol recombinant SARM1 (mol/min/mol SARM1).

### Primary neuronal cultures

Primary cell isolation from C57BL/6Babr and *Sarm1*^-/-^ mice was approved and performed according to regulations of the University of Cambridge under project licence PPL P98A03BF9. Animals were fed ad libitum and held under standard specific pathogen free (SPF) conditions in the mouse facility. Superior cervical ganglia (SCG) were dissected from wild-type or *Sarm1*^-/-^ P1-3 pups following a standardized protocol^49^. Briefly, ganglia were incubated in 0.025% Trypsin (Sigma) in PBS at 37 °C for 20 min followed by 0.2% Collagenase type II (Gibco) in PBS (with Ca^2+^ and Mg^2+^) at 37 °C for 15 min. Subsequent trituration of ganglia was achieved in SCG medium using a pipette tip. Dissociated cells were resuspended in SCG culture medium, comprising Dulbecco's Modified Eagle's Medium (DMEM) (4500 mg/L glucose and 110 mg/L sodium pyruvate, Sigma) supplemented with 2 mM glutamine (Invitrogen), 1% penicillin/streptomycin (Invitrogen), 10% fetal bovine serum (Sigma), 2 μg/ml Aphidicolin (Calbiochem), and 25–50 ng/ml 2.5S NGF (Invitrogen). Dissociated cells were plated on ibidi μ-dishes (Thistle Scientific) coated with poly-L-lysine and Laminin (both Sigma) and fresh SCG medium added every 2–3 days. All methods are reported in accordance with ARRIVE guidelines.

### Microinjections

Microinjections were performed on a Zeiss Axiovert 200 microscope using a Femtojet 5171 transjector with Femtotips and a 5246 micromanipulator system (all Eppendorf). All injection mixes were prepared in 0.5 × PBS(-) and passed through a Spin-X filter (Corning) to avoid needle plugging. The types and concentrations of expression constructs in the injection mixes used are listed in figure legends. The mix was injected into the nuclei of SCG neurons in dissociated cultures and 30–40 SCG neurons were injected per dish (representing one repeat). For experiments in Fig. 4, *Sarm1*^-/-^ SCG neurons were injected after 5–7 days in vitro and axotomy was performed two days after injection. For experiments in Fig. 5, wild-type SCG neurons were injected after 4 days in vitro and the DMEM in SCG medium was replaced with phenol red-free FluoroBRITE medium (Thermo Fisher Scientific) for better visualisation/imaging of neurites expressing relatively low levels of ZsGreen.

### Axon degeneration assay

Neurites were cut manually using a curved scalpel on the second day after injection so that injected soma were severed from the distal ends of their axons. For quantification, numbers of continuous healthy DsRed-labelled axons within the same field were compared distal to the site of a cut at 0 h and 24 h. Fragmented neurites, or those displaying abnormal swelling, were categorized as degenerated. Numbers given on graphs represent the percentage of healthy neurites remaining at 24 h relative to the total number of labelled axons at 0 h after the cut.

### Immunostaining

Expression of exogenous SARM1 variants in microinjected SCG neurons was confirmed by immunostaining. Injected neurons were washed with 1 × PBS, fixed with 4% paraformaldehyde for 20 min at RT, followed by permeabilization with 1% Triton X-100 in PBS for 5 min at RT. Blocking was performed in 50% goat serum in PBS containing 1% BSA for 30 min at RT. Cells were then incubated with the primary antibody (SARM1 monoclonal antibody^54^ 1:500) in PBS, 1% BSA for 1 h at RT. After three washes with PBS, cells were incubated with Alexa Fluor-488-conjugated anti-mouse secondary antibody (1:250) in PBS, 1% BSA for 1 h at RT. Cells were washed three times in PBS, then mounted in Vectashield containing DAPI (Vector Laboratories), for nuclear counterstaining, and coverslips sealed with nail varnish. Images were captured with a Leica DFC365FX fluorescence monochrome 188 camera attached to a Leica DMi8 inverted fluorescence microscope (20 × objective). Laser filter settings were 405 nm and 425/50 for DAPI, 488 nm and 515/30 for AlexaFluor-488 (Sarm1), and 543 nm and 560 LP for DsRed.

### Statistical analysis

Multiple comparisons and multiple paired *t* tests were performed using Prism software (GraphPad Software Inc., La Jolla, CA, USA) as described in the figure legends. Data in graphs are displayed as mean ± standard error of the mean (SEM). Data in Fig. 2a was log-transformed prior to statistical analysis but non-transformed data were plotted for ease of interpretation. Statistical significance was determined using the tests described in each figure legend. A *p* value < 0.05 was considered as significant and values above this cut-off considered not significant (ns). A false discovery rate (FDR) correction was applied to multiple comparisons in all cases and the adjusted *p* value we show represents the q value reported in the test.

## Supplementary Information

Supplementary Information.

## Data Availability

The SARM1 variants identified and characterised in the current study are available in the gnomAD and dbSNP repository, https://gnomad.broadinstitute.org/ and https://www.ncbi.nlm.nih.gov/snp/. Additional data can be made available upon request.
