# Supplementary material for: Natural variants of human SARM1 cause both intrinsic and dominant loss-of-function influencing axon survival
Source: Sci Rep. 2022 Aug 16;12:13846. doi: 10.1038/s41598-022-18052-8 (PMC9381744; doi:10.1038/s41598-022-18052-8)

Supplementary Information

Natural variants of human SARM1 cause both intrinsic and dominant loss-of-function influencing axon survival

Mirlinda Ademi, Xiuna Yang, Michael P. Coleman, and Jonathan Gilley*

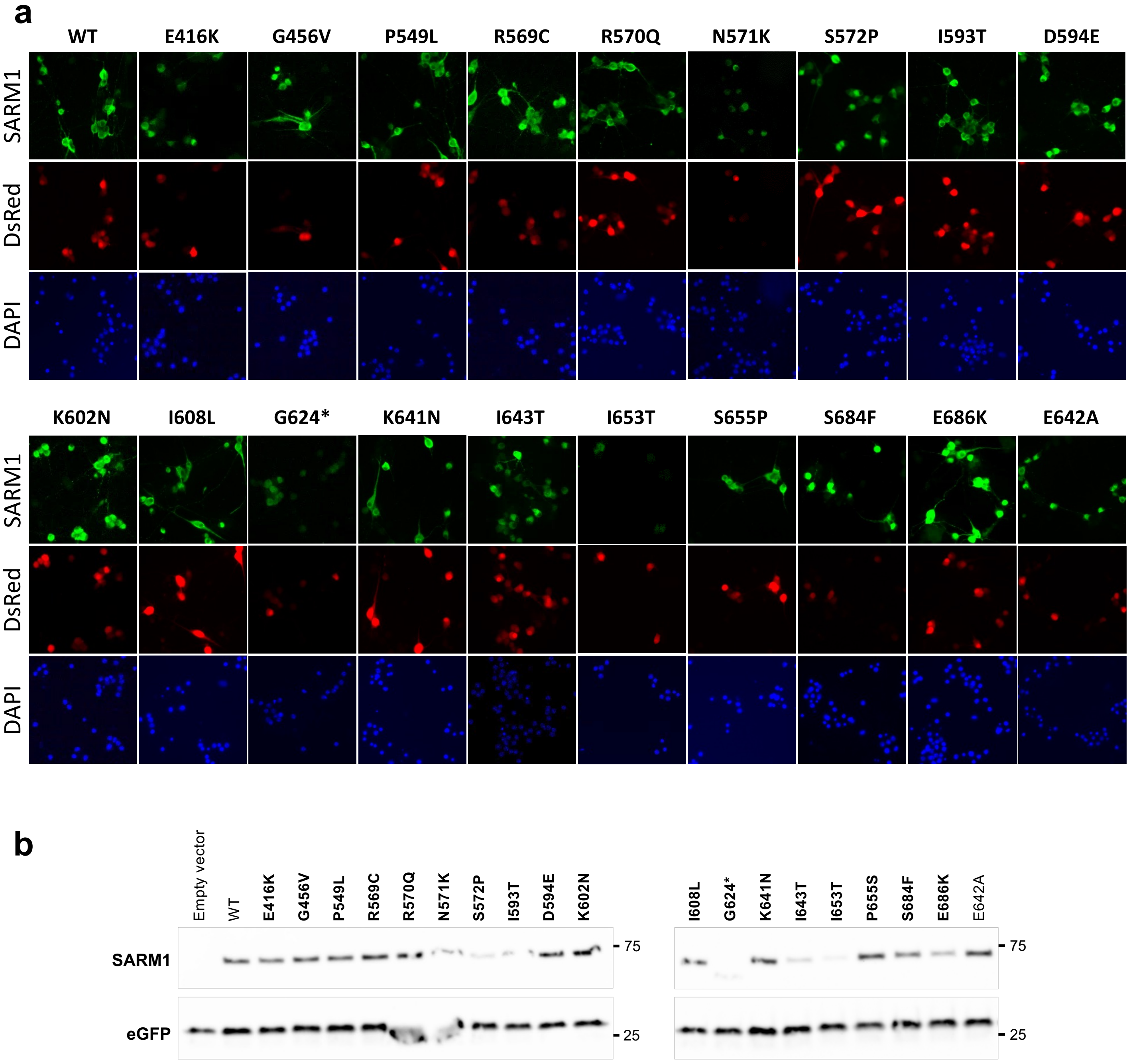

Supplementary Figure 1. Expression of SARM1 variants from pCMV-Tag4-based constructs used for microinjection into SCG neurons.

(**a**) *Sarm1*$^{-/-}$ SCG neurons were co-injected with 25 ng/µl pCMV-Tag4-based expression constructs for untagged WT or variant human SARM1 and 25 ng/µl pDsRed2 encoding DsRed protein. Neurons were immunostained with a SARM1 antibody 24 h after injection, followed by nuclear counterstaining with DAPI. Representative images of SARM1 immunostaining, DsRed fluorescence (as a marker for injected neurons), and DAPI staining are shown for each SARM1 variant, as indicated.

(**b**) Representative immunoblots (of at least n = 4) of extracts of HEK 293T cells 24 h after co-transfection with 0.75 µg pCMV-Tag4-based expression constructs for untagged WT or variant human SARM1 and 0.25 µg pEGFP-C1 encoding eGFP protein (total 1 µg DNA transfected). Blots were probed with antibodies for SARM1 (~70 kDa) and eGFP (~26 kDa, acting as a control for transfection efficiency). All SARM1 variants are expressed and are of the expected size. As in previous experiments (Fig. 2d), G624* SARM1 expression is very low.

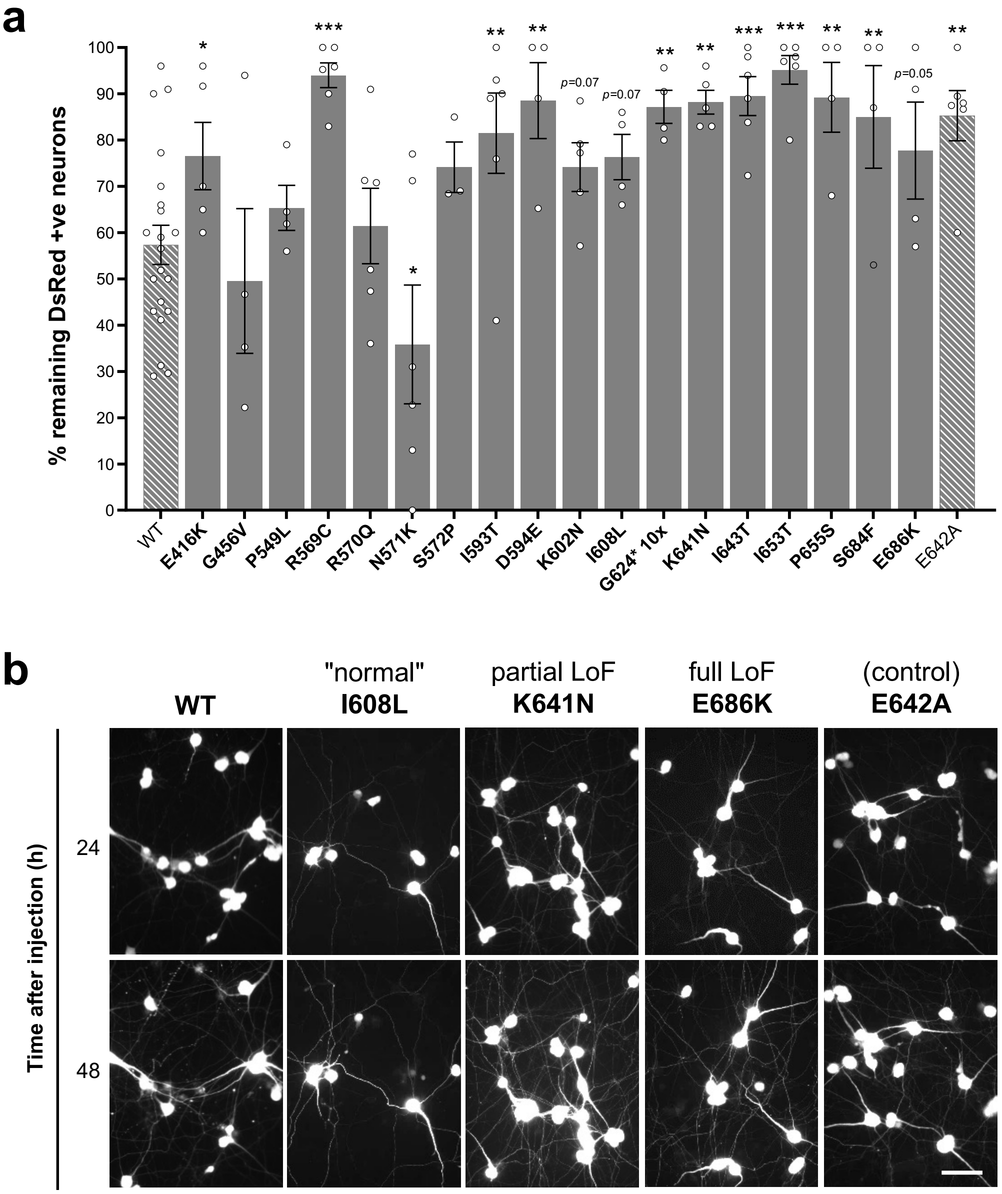

Supplementary Figure 2. Natural human SARM1 variants with a LoF phenotype do not affect cytotoxicity.

(**a**) Survival of the injected *Sarm1*$^{-/-}$ SCG neurons described in Fig. 4A. Loss of injected neurons was determined by comparing cell body size and shape, as revealed by DsRed labelling, at 24 h and 48 h after injection, as has been described previously (Gilley & Coleman, 2010). The number of morphologically normal cell bodies at 48 h post microinjection is shown as a percentage of the total number of those present at 24 h. Means ±SEM and individual data points (n = 3-20) are plotted. * $p < 0.05$, ** $p < 0.01$, *** $p < 0.001$, multiple pairwise comparisons to WT SARM1 with a FDR correction.

(**b**) Representative fluorescence images, as used for the quantification shown in part **a**, of microinjected *Sarm1*$^{-/-}$ neuronal cell bodies injected with constructs expressing WT or variant/mutant SARM1 (as indicated) at 24 and 48 h after injection. As in Figure 4, controls (wild-type and enzyme-dead E642A SARM1) and three exemplary phenotypes are shown: I608L SARM1 resembling wild-type SARM1 ("normal"), K641N SARM1 displaying a partial LoF phenotype, and E686K SARM1 displaying full LoF. Scale bar, 50 μm.

Uncropped immunoblot images for Figure 2D

Top panels:

Top half of membrane probed for Flag (SARM1)

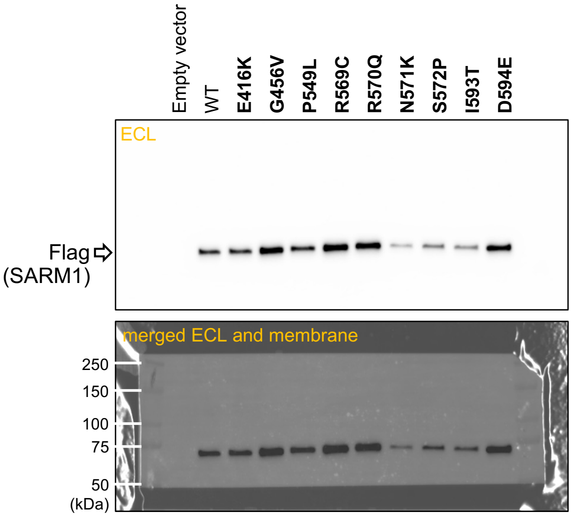

Bottom half of membrane probed simultaneously for GAPDH and ZsGreen

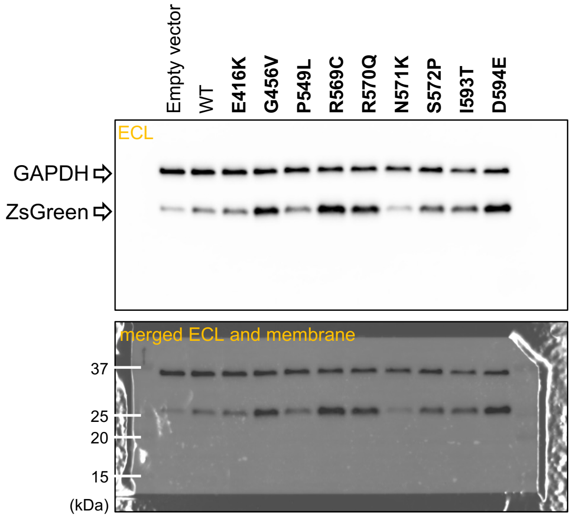

Bottom panels:

Top half of membrane probed for Flag (SARM1)

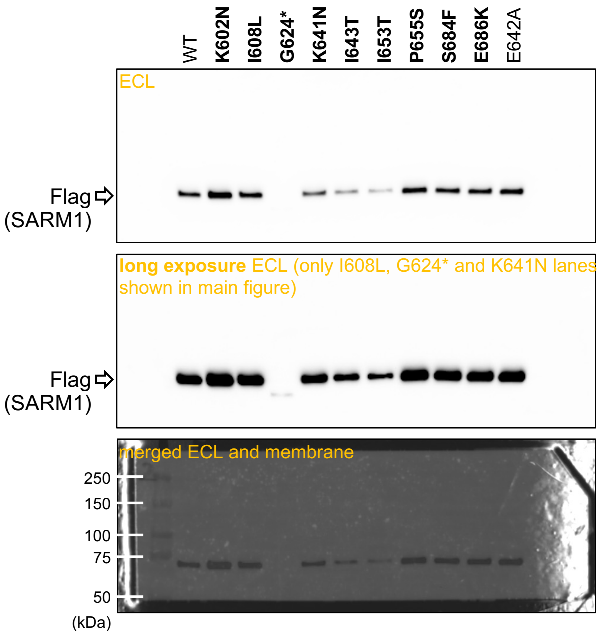

Bottom half of membrane probed sequentially for GAPDH and ZsGreen

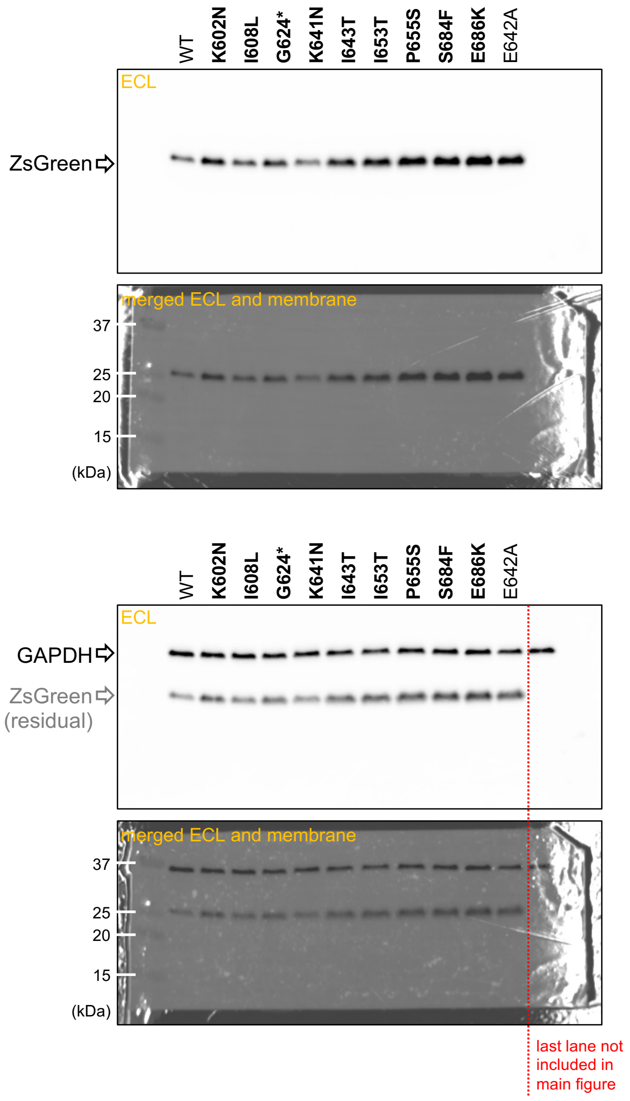

Uncropped immunoblot images for Supplementary Figure 1B

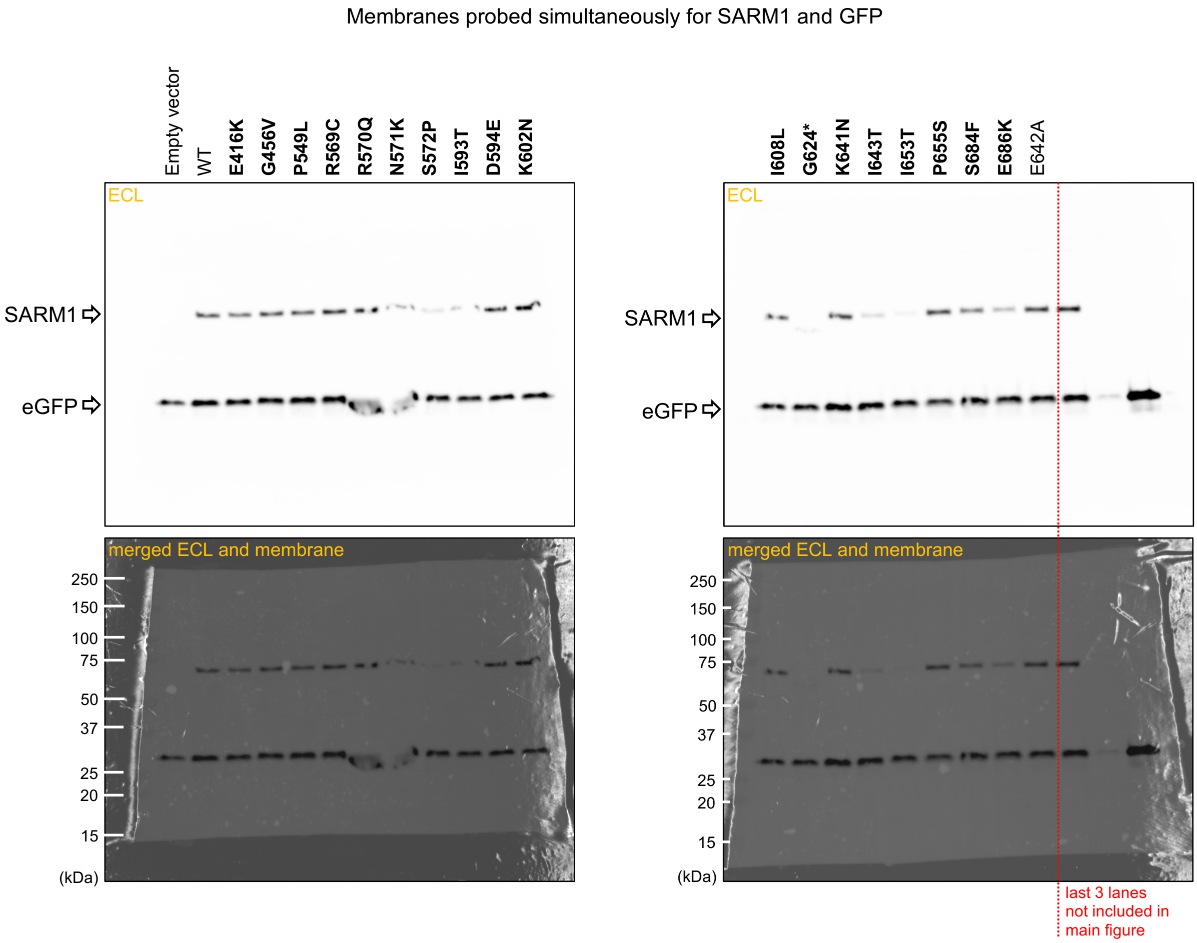

Supplement: Supplementary file 1 — Supplementary Information. [file 41598_2022_18052_MOESM1_ESM.pdf]
